# Great Achievements under Suffering

**Published:** 1893-04-29

**Authors:** 


### Great Achievements under Suffering.

### I.—PROFESSOR HENRY FAWCETT.

Looking into the records of those who have done great things in life a strange power of compensation in nature is revealed. The battle is not always to the strong, nor the race to the swift. The energy diverted from one channel rushes with redoubled impetus to carve out for itself another, and talents apparently buried fathoms deep have been made to yield rich usury to the Giver. Very full of encouragement to suffering humanity is the record of such lives. No bodily infirmity, no oppression even of mind, has proved in the world's history an invincible bar to service for man. From the days of Job, the first invalid poet, to the days of the maimed hero Kavanagh, the glory of the fiery spirit has been sufficient to ennoble the tenement of clay, supplying with infinite resource the place of forfeited sense or limb, and dispensing miraculously with those common gifts of health and strength on which men are accustomed to rely for powers of work. The brief story of some such lives we propose to place before our readers—to the strong a lesson of humility in the presence of human suffering; to the weak a trumpet call to triumph over the tyranny of circumstance.

In one form or another the tyranny of circumstance has to be met and overcome in every life. Poverty, obscurity, the opposition of authority, the claims of family life, or even the vexatious cords of rank and wealth—any one of these may serve just as surely as physical infirmity to test a vocation and develop hidden strength by opposition. But the temptations to succumb are increased tenfold when the difficulties start from within instead of from without. When self has to be moulded instead of the world a crowd of reasons make themselves heard why the struggle should be abandoned. The very kindness of friends contributes to inaction, till the will itself is sapped, and the condition is reached, noted by Solomon as saddest of all the pains of old age, when desire shall fail.

In such a mood, the story of Professor Fawcett's life, as told by his biographer, Mr. Leslie Stephens, is one to stimulate resistance to every form of morbid surrender to what men lightly call the inevitable. Born at Salisbury in 1833, he was educated at King's College School and Trinity Hall, Cambridge, and till his twenty-fifth year enjoyed every advantage of health and opportunity to fit him for his chosen career, "that of exerting an influence in the House of Commons in removing the social evils of our country, and especially the paramount one—the mental degradation of millions." In 1858, while out with a shooting party, two pellets from a charge aimed by his father, whose sight was defective, passed through the tinted glasses he was wearing at the time and deprived him instantly of sight for ever. During the miserable period of prostration and inaction which followed it began to be assumed by all his friends that all hopes of a useful career were at an end. Resignation was the theme of every sympathizer. The steadfast purpose which led him (so he afterwards said) to determine within ten minutes of the accident to stick to his old pursuits, lay for a time dormant. It was revived by a stirring letter from his old tutor, recalling him from endurance to action. "The evil that has fallen upon you, like all other evils, will lose half its terrors if regarded steadfastly in the face with the determination to subdue it as far as it may be possible to do so." From henceforth he determined not that he would in some way evade, but that he would conquer his fate. From the highest of all reasons, care for the poor and helpless, he had aspired to distinction in a public career. That career he resolved still, in the teeth of every difficulty, to pursue.

It is not always remembered that every great resolve is made up in practice of unending attention to detail. Much of Fawcett's success in great matters can be directly traced to the unfailing resource and patience with which he met the countless difficulties presented by even the simplest occupations. To write books and make speeches which should help to mould the thoughts of millions lay in the dim future. The present was made up of continual efforts to escape the tedium of inaction, and live, instead of enduring life. And it must be remembered that Fawcett was wanting in some of the special gifts which seem partly to atone in some cases for want of sight. Music was nothing to him at the time of the accident, and though he deliberately cultivated the taste, it was only in later years that it became a principal source of enjoyment. The dexterity of touch too, and accurate verbal memory which characterises people who early lose their sight, were never his. His want of manual skill was replaced by his fearless good humour over his own blunders, and his generous reliance on the good will of every one about him. This never failed him. Speaking on February 18th, 1880, he says: "The chief compensation, the silver lining to the dark cloud, is the wonderful and inexhaustible fund of human kindness to be found in this world; the appreciation which blind people must have at every moment of their life, of the cordial and ready willingness with which the services which they needed were generously offered them." Nothing was of greater service to him in his public life than this happy temper of mind, the result no less of continual self-discipline in daily trifles than of natural disposition. In dealing with large and often unfriendly crowds in his early unsuccessful contests, this capacity for taking hold of men by the right handle was his surest gift, and the confidence in his audience which showed in his whole bearing more than compensated for the want of power to watch the effect of his words.

It has been said that he was wanting in accurate verbal memory. Like Charles Lamb, he seldom achieved a quotation correctly, unless for speech-making purposes he learnt a passage laboriously by heart. But this deficiency, again, was turned to advantage by the cultivation of his naturally vivid power of picturing scenes and of forming and modifying impressions. His marvellous grasp of statistics was undoubtedly due to this picturing power. He saw rather than remembered the various groups of figures which he was able to manipulate with a dexterity which roused amazement in his colleagues. He could comment in all sincerity on the improved appearance of a friend's house, and startle his hearers by observations on the altered looks or odd gestures of other men. In the same way, it needed only a word or two from the companion of his walks to bring the scenery vividly before him, so vividly indeed, that he once said that in after recollections of such scenes he had to check himself and consider for a moment whether the impression was produced when he had his sight or was conveyed by the description of another.

In this way one might almost say he built up new senses to replace the one he had lost. In his determination to stick to his old pursuits he acquired from the very first a habit of fearless active exercise. In walking, rowing, riding, and skating few could equal him, and in fishing he learnt to find one of his most valuable forms of relaxation.

The strength of will exercised over these seemingly unimportant matters was needed to the full when his real difficulties began in the opening of his political career. In his devotion to the problems of political economy, he may have found his blindness less a drawback than an incentive. As his biographer says: "Blindness increased concentration by shutting out distractions. We close our eyes to think, and his were always closed." He had been "an economist from infancy," but it was only after his loss of sight that these questions engrossed him heart and soul. He soon began to be known among a few as a thinker and a writer, but even these few were against him in his fixed resolve to pursue the active career of his choice. No one but himself believed in the possibility of his success. His most intimate friends and admirers considered "it was really a kindness to intimate quietly, but firmly, that his ambition was illusory, and coax him back to his study to amuse himself with speculations on abstract politics." It was done in the end by sheer force of will, for he had neither influence nor money to aid in the struggle. In the face of every possible discouragement he contested three seats—Southwark, Cambridge, and Brighton—unsuccessfully. His first public success was achieved when, in 1863, he was elected Professor of Political Economy at Cambridge, after an exciting contest. Two years later he was elected member for Brighton, and thus seven years after his great calamity gained the object of his immediate ambition. The history of his after career is still fresh in the minds of everyone. Always the application of the same principles which guided and stimulated him in the first years of struggle can be noticed. And it is good to be reminded again and again by his biographer that pre-eminently in his own life the words which he spoke once to his fellow-sufferers proved true in the fullest sense: "That for every blind man there was still good work to do, and the more active his career, the more useful his life to others, the more happy his days to himself."

